# Analysis of risk factors for massive intraoperative bleeding in patients with placenta accreta spectrum

**DOI:** 10.1186/s12884-022-04391-x

**Published:** 2022-02-11

**Authors:** Yuanyuan Wang, Yadan Zhou, Lin Zeng, Lian Chen, Yangyu Zhao

**Affiliations:** 1 Department of Obstetrics and Gynecology, Peking University Third Hospital, Beijing, 100191 China; 2 National Clinical Research Center for Obstetrics and Gynecology, Peking University Third Hospital, Beijing, 100191 China; 3 Department of Obstetrics and Gynecology, Zhengzhou Central Hospital, Zhengzhou, 450007 Henan China; 4 Research Centre of Clinical Epidemiology, Peking University Third Hospital, Beijing, 100191 China

**Keywords:** Placenta accrete spectrum, Hemorrhage, Ultrasonography, Cesarean section, Hysterectomy

## Abstract

**Background:**

To analyze relevant factors for massive postpartum hemorrhage in women with placenta accreta spectrum in order to improve the ability to identify those at risk for intraoperative bleeding and improve outcome.

**Methods:**

This study is a retrospective study and based on data from Hospital electronic medical record. Placenta accreta patients who delivered by cesarean section at Peking University Third Hospital from September 2017 to December 2019 were selected and included. According to the amount of intraoperative bleeding, they were categoried into the massive bleeding group (bleeding volume ≥ 2000 mL, 68 cases) and non-massive bleeding group (bleeding volume < 2000 mL, 99 cases). Univariate analysis and multivariate logistic regression were used to analyze the correlations between related risk factors or ultrasound imaging characteristics and the severity of bleeding during operation.

**Results:**

(1) There were statistically significant differences in gravidity, parity, number of prior cesarean deliveries and placenta accreta ultrasound scores (*P <* 0.05) between the two groups of patients.

(2) Among the ultrasonographic indicators, the disappearance of the post-placental clear space, the emergence of cross-border blood vessels in the region of subplacental vascularity, interruption or disappearance of the bladder line, and the presence of the cervical blood sinus had the most significant correlation with hemorrhage during PAS (*P <* 0.05).

**Conclusion:**

The presence of cervical blood sinus, interruption or disappearance of bladder line, the disappearance of the post-placental clear space and abnormal subplacental vascularity are independent risk factors for massive hemorrhage during PAS. We should pay more attention to these indicators in prenatal ultrasound examination in order to reduce the intraoperative bleeding and improve maternal outcomes.

## Background

Placenta accreta spectrum (PAS) is a serious life-threatening obstetric disorder that refers to an abnormal placental attachment caused by the invasion of placental villi into the myometrium. According to the depth of placental invasion into the myometrium and degree of infiltration into the organs adjacent to the uterus, the following condition of abnormal invasive placenta can occur: (1) placenta accreta, in which the placenta invades the superficial myometrium of the uterus; (2) placenta increta, in which the placenta invades the deep myometrium of the uterus; and (3) placenta percreta, in which the placenta penetrates the uterine wall and reaches the serous layer of the uterus and even invades the organs adjacent to the uterus [1].

Observational studies have shown that the prevalence of PAS was between 1 in 2,510 and 1 in 4,017 from the 1970s to 1980s, in contrast to the rate of 1 in 533 observed from 1982 to 2002 [2–4]. Another study using the 1998 - 2011 National Inpatient Sample found that the overall rate of PAS in the United States was 1 in 272 [5]. With the implementation of the "two-child” policy in China, the proportion of pregnant women with a scarred uterus who become pregnant again is increasing rapidly. The incidence of complications associated with a scarred uterus, such as pernicious placenta previa and PAS, is also on the rise. As a maternal referral center, the Obstetrics Department of the Third Hospital of Peking University is responsible for the management of severe cases, including PAS cases. As a result, the rate of PAS in our hospital increased from 0.1% to 3.4% during the period from 2007 - 2019 among women who had a discharge diagnosis.

The major adverse outcomes of PAS include significant postpartum hemorrhage (PPH) and high hysterectomy rates among women of reproductive age. PPH is a leading cause of maternal morbidity and mortality worldwide. The average blood loss of those patients with placenta increta/percreta during emergency surgery is up to 8600 mL [6]. Bailit JL et al conducted a study showing that the median estimated intraoperative blood loss of PAS was 2 L [7]. Therefore, prenatal diagnosis and proper perioperative management for PAS are crucial. At present, ultrasound examination and, especially, magnetic resonance imaging (MRI) are the main methods for the prenatal diagnosis of PAS. Predicting the degree of malignancy in PAS through ultrasound, ensuring adequate preoperative preparation and guiding timely referral by primary hospitals are of great significance for reducing the serious complications of PAS.

Therefore, our team established an “Placenta Accreta Spectrum Ultrasound Scoring System” (PASUSS) (Table 1) that could estimate the severity of PAS in 2016. However, in clinical practice, the difference in intraoperative blood loss among those who are considered to have the same disease classification before delivery is sometimes large. Determining the risk factors related to massive bleeding before operation and how to take preventive measures in advance to reduce intraoperative bleeding are difficult problems to solve at present. Most current PAS studies focus on high-risk factors, prediction of severity, and multidisciplinary management. However, there are few analyses related to risk factors for massive intraoperative bleeding. Therefore, this study retrospectively analyzed the clinical features and related risk factors for massive PPH during the operation to provide a reference for the prevention and treatment of massive bleeding during the operation, which is better to reduce the blood transfusion rate and improve maternal outcomes [8].

**Table 1:** Placenta Accreta Spectrum Ultrasound Scoring System

	**0**	**1**	**2**
**Position of the placenta**	Normal	Marginal placenta previa or low-lying placenta	Completely placenta previa
**Thickness of the placenta**	<3 cm	3–5 cm	>5 cm
**Continuity of the clear space**	Continuity	Local interruption	Disappeared
**Bladder line**	Continuity	Local interruption	Disappeared
**Lacuna**	None	Present	Fused with boiling water sign
**Condition of the subplacental vascularity**	Normal blood flow	The blood flow increased, forming a cluster	“cross-border” blood vessels
**Blood sinus of cervix**	None	Present	Fused with boiling water sign
**Morphology of cervix**	Complete	Incomplete	Disappeared
**Number of prior cesarean deliveries**	0	1	≥ 2

## Methods

### Sources of patients

PAS patients who were admitted to Peking University Third Hospital for cesarean section delivery from September 2017 to December 2019 were selected for in-depth analysis. The inclusion criteria were as follows: (1) single pregnancy; (2) gestational age ≥ 28 weeks; (3) ultrasound scoring that was performed in our hospital before delivery to predict PAS and its type, with an ultrasound score ≥ 6 points; (4) PAS as the operative indication of elective cesarean section; and (5) clinically or pathologically confirmed PAS after operation. If pathological examination was performed, then diagnosis could be combined with pathology. The exclusion criteria were as follows: (1) patients without complete data; (2) patients who were evaluated preoperatively and eventually underwent hysterectomy of the placenta in its original position; (3) patients with intraoperative abdominal aortic balloon placement and (4) patients with abnormal coagulation or primary uterine contractions. A total of 167 patients with PAS were enrolled according to the above criteria. All selected patients underwent multidisciplinary management before surgery, and individualized surgical procedures were developed. All operations were performed by a team of senior obstetricians.

### Study design

Based on clinical experience and relevant literature information, the patient information collected included maternal age, gravidity, parity, miscarriage history, number of prior cesarean deliveries, pre-delivery PASUSS scores and imaging characteristics, gestational weeks at delivery, intraoperative blood loss, and hysterectomy. All patient information was retrieved from the hospital’s electronic medical record system. The study was approved by the hospital ethics committee, and the committee approval number is 2015–155–2. All patients were informed regarding the relevant matters before study initiation. Patients voluntarily entered the study and signed informed consent forms.

### Clinical data collection and examination method

PPH is now defined as a blood loss of 1000 mL or more or signs or symptoms related to hypovolemia that occurs within 24 hours after delivery, regardless of the mode of delivery[9]. According to the amount of intraoperative blood loss, 68 patients with blood loss ≥ 2000 mL were included in the massive hemorrhage group (MHG) and 99 patients in the non-massive hemorrhage group (NMHG) with bleeding less than 2000 mL. Blood loss was evaluated by the volumetric and weighing methods.

There are 9 scoring items recognized in the literature as indicators in the “PASUSS” for diagnosing PAS (Table 1): placenta position, placental thickness, continuity of clear space, bladder line, lacuna, condition of subplacental vascularity, blood sinus of cervix, cervical morphology, and a history of cesarean section also remains essential for diagnosing PAS. All 9 items were rated as 0, 1, and 2 points depending on the options, while the sum of the scores reflected the possible severity of PAS. The higher the score, the higher the severity of the disease. A score of 3 to 5 points indicated accreta; 6 to 9 points indicated increta; and 10 or more points indicated percreta.

The color Doppler ultrasound—Philips Iu22 (made by Philips Ultrasound, Inc. in Bothell Everett Highway Bothell, WA) and GE Volusion E8 (made by GE Healthcare Austra Gmbh in Tiefenbach 15, 4871 Zipf, Germany) were used for examination. The probe frequency was 3.5 Hz. Per routine, patients were initially placed in the supine position and lateral position when necessary[8]. The above patients were examined by ultrasound at different gestational weeks. We scored every patient according to the scoring system presented in Table 1 and collected their clinical data.

### Statistical methods

Statistical analysis was performed with SPSS 26.0 software. The enumeration data are expressed as the number of cases and percentage (%), and the χ2 test was used for the comparison of enumeration data between groups. Unconditional binary logistic regression was used to analyze the risk factors for bleeding during severe PAS the backward LR was used to establish a multivariate analysis model, and variables with *P <* 0.10 were included in the final model. The odds ratio (OR), 95% confidence interval (95% CI), and correlation between related factors and the degree of massive bleeding during PAS were calculated, and *P <* 0.05 indicated statistically significance.

## Results

3.1. A total of 167 cases were enrolled, including 99 cases in the NMHG and 68 cases in the MHG. The ages were 33.0 ± 4.8 years in the NMHG and 33.5 ± 3.9 years in the MHG. There were 81 (81.8%) and 66 (97.1%) multiparous mothers in the NMHG and MHG, respectively, and 18 cases (18.2%) in the NMHG and 22 cases (32.4%) in the MHG had a history of more than 2 cesarean sections. In the NMHG, 88 cases (88.9%) had complete placenta previa, while the MHG group had 65 such cases (95.6%). Regarding those last PASUSS score was ≥ 10 points, there were 27 cases (27.3%) and 56 cases (82.4%) in the NMHG and MHG, respectively. The gestational week at delivery in the NMHG was 35.6 (34.6 - 37.0) weeks, which was greater than 34.7 (33.3 - 36.0) weeks in the MHG. The intraoperative blood loss of the two groups was 800 (600-1400) mL in the NMHG and 3000 (2300 - 4000) mL in the MHG. The hysterectomy rate among patients in the MHG was 44.1% (30/68), which was significantly higher than the rate of 2.0% in the NMHG (2/99).

There were statistically significant differences in gravidity, parity and number of prior cesarean deliveries (*P <* 0.05) between the two groups. In the MHG, the ultrasound score was ≥ 10 points, and the rate of hysterectomy was significantly higher than that in NMHG (*P <* 0.05). There was no significant difference in age or number of abortions (*P* > 0.05). The general information of the patients is presented in Table 2.

**Table 2:** Comparison of the general conditions of the two groups of patients

Variable	NMHG (*n*=99)	MHG (*n*=68)	χ^2^	*P-value*
Maternal age (years)				
<35	63(63.6%)	42(61.8%)	0.060	0.806
≥35	36(36.4%)	26(38.2%)		
Gravity				
≤2	31(31.3%)	11(16.2%)	4.906	0.027
>2	68(68.7%)	57(83.8%)		
Parity				
0	18(18.2%)	2(2.9%)	11.252	0.004
1	62(62.6%)	43(63.2%)		
≥2	19(19.2%)	23(33.9%)		
Number of abortions				
0	34(34.3%)	16(23.5%)	3.722	0.156
1	27(27.3%)	16(23.5%)		
≥2	38(38.4%)	36(52.9%)		
Number of prior cesarean deliveries				
0	21(21.2%)	4(5.9%)	9.717	0.008
1	60(60.6%)	42(61.8%)		
≥2	18(18.2%)	22(32.4%)		
PASUSS Score				
<10	72(72.7%)	12(17.6%)	48.921	<0.001
≥10	27(27.3%)	56(82.4%)		
Hysterectomy	30(44.1%)	2(2.0%)	46.120	<0.001

### Analysis of related ultrasound imaging characteristics of the two groups of patients

Compared with that in the NMHG, the incidences of the 6 ultrasound characteristics, namely, disappearance of continuity of the clear space, interruption or disappearance of the bladder line, placental lacuna with a boiling water sign, “cross-border” blood vessels in the subplacental vascular region, a cervical sinus and abnormal cervical morphology, were significantly higher in the MHG. The differences were statistically significant (*P <* 0.05). However, there was no significant difference in complete placenta previa or placental thickness ≥ 5 cm (*P* > 0.05, see Table 3 for details).

**Table 3:** Comparison of ultrasound imaging characteristics between the two groups

Variable	NMHG(*n*=99)	MHG(*n*=68)	χ^2^	*P*-value
Complete placenta previa				
NO	11 (11.1%)	3 (4.4%)	2.356	0.125
YES	88 (88.9%)	65 (95.6%)		
Tickness of the placenta≥5cm				
NO	70 (70.7%)	39 (57.4%)	3.171	0.075
YES	29 (29.3%)	29 (42.6%)		
Continuity of the clear space disappeared				
NO	79 (79.8%)	27 (39.7%)	27.947	<0.001
YES	20 (20.2%)	41 (60.3%)		
Bladder line interrupted or disappared				
NO	74 (74.7%)	27 (39.7%)	20.709	<0.001
YES	25 (25.3%)	41 (60.3%)		
Lacuna fused with boiling water sign				
NO	54 (54.5%)	22 (32.4%)	8.006	0.005
YES	45 (45.5%)	46 (67.6%)		
“cross-border” blood vessels of the subplacental vascularity				
NO	66 (66.7%)	19 (27.9%)	25.687	<0.001
YES	32 (32.3%)	49 (72.1%)		
Cervical sinus				
NO	68 (68.7%)	19 (27.9%)	26.817	<0.001
YES	31 (31.3%)	49 (72.1%)		
Abnormal cervical morphology				
NO	85 (85.9%)	48 (70.6%)	5.797	0.016
YES	14 (14.1%)	20 (29.4%)		

### Multivariate analysis of general condition, ultrasound imaging characteristics and degree of bleeding during PAS in the two groups

The statistically significant general conditions and ultrasound imaging characteristics of the two groups of patients were analyzed by unconditional binary logistic regression analysis. The disappearance of the post-placental clear space (OR 3.87; 95% CI 1.82 - 8.24), the emergence of cross-border blood vessels at the subplacental vascular region (OR 2.42; 95% CI 1.08 - 5.44), interruption or disappearance of the bladder line (OR 2.41; 95% CI 1.07 - 5.42) and a cervical sinus (OR 2.26; 95% CI 1.08 - 4.75) were 4 ultrasound imaging features that were independent risk factors for massive PPH during operation (*P <*0.05, see Table 4).

**Table 4:** Logistic regression analysis of high-risk factors for PPH of severe placenta accreta

Variable	OR	95%CI	*P*-value
Continuity of the clear space disappeared			
NO	1.00		
YES	3.87	1.82-8.24	<0.001
“cross-border” blood vessels of the subplacental vascularity			
NO	1.00		
YES	2.42	1.08-5.44	0.033
Bladder line interrupted or disappared			
NO	1.00		
YES	2.41	1.07-5.42	0.034
Cervical sinus			
NO	1.00		
YES	2.26	1.08-4.75	0.031

## Discussion

### The need for prenatal prediction of massive bleeding during PAS

Previous cesarean sections and placenta previa are the most common risk factors for PAS. Other forms of uterine surgery, including myomectomy, uterine curettage, and hysteroscopic surgery, are also risk factors for PAS, although it is difficult to ascertain an absolute risk. Prior endometrial ablation, pelvic irradiation, and assisted reproductive technology are also risk factors for developing PAS [1, 10]. The incidence of PAS in patients with a history of cesarean section accompanied by placenta previa was much higher than that in patients with prior cesarean section but without placenta previa. When the numbers of cesarean sections were 1, 2, 3, 4, 5 and ≥6, the incidences of PAS were 3.3%, 11%, 40%, 61%, 67%, and 67%, respectively, in the MHG and 0.03%, 0.2%, 0.1%, 0.8%, 0.8% and 4.7%, respectively, in the NMHG. With increasing number of prior cesarean sections, the incidence of postpartum hemorrhage in patients with PAS tends to increase [4, 11–13]. This association is thought to be due to poor repair of the endometrium and/or decidua basalis and the relative hypoxia of cesarean scar tissue (caused by fibroblast repair and a decreased vascular concentration). During subsequent gestation, cytotrophoblasts invade the decidualized endometrium but do not enter the spongiosus layer nor do they encounter normal signals to prevent invasion. Instead, trophoblast cells continue to invade to an abnormal degree. Histopathological assessment of PAS specimens supports this theory [14]. This may be one of the causes of postpartum hemorrhage. As mentioned, PAS can result in a life-threatening hemorrhage, and the median blood loss is reported to be high-volume blood loss from 2000 to 7800 mL [7, 15].

In this study, the incidences of cesarean section and complete placenta previa in the MHG were 94.2% and 95.6%, respectively, and these rates were significantly higher than those in the NMHG (78.8% and 88.9%). The average volume of blood loss in the MHG was 3000 mL, including 7 cases (10.3%) with 5000 - 7900 mL, 2 cases (2.9%) with 8000 - 10000 mL, and 1 case up to 11000 mL. This was significantly higher than the 800 (600 - 1400) mL of blood loss in the NMHG. Rebonato et al reported that intraoperative blood loss was 3340 ± 1264 mL and that the total hysterectomy rate was 66.70%[16]. Our data showed that the hysterectomy rate of patients in the MHG group was 44.1%, which was significantly higher than that of the patients in the NMHG group (2.0%). Hysterectomy is one of the main surgical methods for PAS, especially for percreta. Although it can reduce intraoperative bleeding and the occurrence of complications, it places a great psychological burden on patients and their families. Reducing intraoperative bleeding while preserving the uterus is the main problem encountered by obstetricians. Therefore, it is necessary to identify the risk factors for intraoperative hemorrhage before delivery and actively take preventive measures, as this would have significant clinical impact on reducing perioperative complications.

### Correlation between ultrasound signs and massive blood loss during PAS

In recent years, ultrasound examination, as the primary method for prenatal diagnosis of PAS, has mainly relied on the subjective interpretation of the "typical" signs by two-dimensional grayscale ultrasound and color Doppler ultrasound. Some parameters were analyzed by meta-analysis, which showed that the sensitivity and specificity of ultrasound for the prenatal diagnosis of PAS were > 90% respectively [17, 18]. Most of the current ultrasound studies are based mainly on how to diagnose PAS. There are few reports using the ultrasound scoring scale to predict PAS before delivery and even fewer studies on the predictive ability of various ultrasound indexes for intraoperative blood loss and hysterectomy. The PASUSS used in this article has been used in dozens of hospitals in China. It provides a simple and practical evaluation tool for early detection and timely referral of PAS patients in primary hospitals and provides sufficient and efficient multidisciplinary preparation for the perioperative period. It provides a theoretical basis to most effectively guarantee maternal safety and balance medical resources. The higher the score is, the higher the risk of intraoperative bleeding and hysterectomy will be. However, it has also been found in clinical practice that although the degree of PAS in imaging examination is heavier, the amount of intraoperative blood loss may not be much; different PAS cases with the same total score sometimes also have greater differences in intraoperative blood loss. This study found that the disappearance of the postplacental clear space, the emergence of cross-border blood vessels at the subplacental vascular region, the interruption or disappearance of the bladder line, and a cervical sinus were independent risk factors for massive bleeding in PAS. Studies have shown that the disappearance of the hypoechoic band after the placenta indicates that the predictive accuracy of PAS is lower than that of other signs, but it may indicate a higher risk of intraoperative bleeding [19]. Comstock believes that the interruption of the "bladder line" is caused by the increase in blood vessels at the interface, and the increase in abnormal blood vessels can cause intraoperative bleeding. Abnormal blood flow at the base of the placenta is the formation of abundant new blood vessels at the uterine-placental interface. Ultrasound shows that the structure of the placenta and the uterus or the placental interface is disordered, the blood vessels are dilated, and the blood flow signals are abundant, even reaching the myometrium, which can cause massive bleeding [20, 21]. In addition, the second evaluation of the depth of placental villus implantation into the myometrium and the selection of appropriate surgical methods are also very important for reducing intraoperative bleeding.

## Conclusions

PAS is the main cause of massive postpartum hemorrhage and perinatal hysterectomy. Thus, it is necessary to identify the risk factors for massive hemorrhage in PAS during the prenatal period and make full preparation for the perioperative period, as this is of great clinical significance for reducing the surgical risk and improving the prognosis.

## Data Availability

The datasets generated and analysed during the current study are not publicly available due to patient privacy but are available from the corresponding author on reasonable request.

## Abbreviations

PAS: placenta accreta spectrum
PPH: postpartum hemorrhage
PASUSS: Placenta Accreta Spectrum Ultrasound Scoring System
MHG: massive hemorrhage group
NMHG: non-massive hemorrhage group
OR: odds ratio
CI: confidence interval
